# ncDNA and drift drive binding site accumulation

**DOI:** 10.1186/1471-2148-12-159

**Published:** 2012-08-30

**Authors:** Troy Ruths, Luay Nakhleh

**Affiliations:** 1 Department of Computer Science, Rice University, TX, Houston, USA

## Abstract

**Background:**

The amount of transcription factor binding sites (TFBS) in an organism’s genome positively correlates with the complexity of the regulatory network of the organism. However, the manner by which TFBS arise and accumulate in genomes and the effects of regulatory network complexity on the organism’s fitness are far from being known. The availability of TFBS data from many organisms provides an opportunity to explore these issues, particularly from an evolutionary perspective.

**Results:**

We analyzed TFBS data from five model organisms – *E. coli K12*, *S. cerevisiae*, *C. elegans*, *D. melanogaster*, *A. thaliana* – and found a positive correlation between the amount of non-coding DNA (ncDNA) in the organism’s genome and regulatory complexity. Based on this finding, we hypothesize that the amount of ncDNA, combined with the population size, can explain the patterns of regulatory complexity across organisms. To test this hypothesis, we devised a genome-based regulatory pathway model and subjected it to the forces of evolution through population genetic simulations. The results support our hypothesis, showing neutral evolutionary forces alone can explain TFBS patterns, and that selection on the regulatory network function does not alter this finding.

**Conclusions:**

The *cis*-regulome is not a clean functional network crafted by adaptive forces alone, but instead a data source filled with the noise of non-adaptive forces. From a regulatory perspective, this evolutionary noise manifests as complexity on both the binding site and pathway level, which has significant implications on many directions in microbiology, genetics, and synthetic biology.

## Background

Binding sites are short DNA sequences to which transcription factors bind and regulate gene expression. Their accurate detection would shed light on the process of transcription regulation, which is an essential component of the central dogma of molecular biology. However, due to their short length, which is generally between five and twenty base pairs, binding sites are hard to detect reliably based solely on sequence analysis [1]. Recent advances in biotechnologies, such as ChIP-seq and ChIP-chip, are enabling *in vivo* identification of binding sites, along with their affinity to transcription factors (TF), across entire genomes with high accuracy [2].

The availability of these data allows for new analyses and investigations into the role of transcription regulation in cellular decision making and, indeed, the entire functioning of an organism. For example, the location and TF affinity of binding sites have been used to calculate properties of the regulatory network [3]. Such properties include the connectivity of a network (degree), the robustness of a network to edge manipulation (redundancy), and the robustness of regulatory sequence to mutations (multiplicity). In addition to informing about the topology of regulatory networks, they also have functional implications [4]. From a developmental perspective, numerous studies have shown that regulation of gene expression has either accompanied or even facilitated complexity in developmental systems, driven by the accumulation and proliferation of both transcription factors and their binding sites [5-10]. We therefore use these properties — degree, redundancy, multiplicity, and number of binding sites — to measure the binding site accumulation and its effect on regulatory complexity across a diverse group of organisms. Because these properties report on the density of the network at both the pathway and sequence level, we use them as a proxy for regulatory complexity.

Additionally, the availability of genomic data, along with annotations of functional features, is providing an opportunity for exploring the interplay between evolutionary forces and the amount of regulatory regions including binding sites. For example, a recent study comparing human and chimpanzee genomes revealed more signatures of adaptation, or rapid change, in regulatory regions than in coding regions [11]. Further, recent analyses have shown that conserved non-coding DNA (ncDNA), which harbors binding sites, is almost three times the amount of conserved protein-coding DNA in the *Drosophila* genome [12]. Other studies have shown that the amount of conserved ncDNA underestimates the amount of ncDNA with functional roles [13,14]. In fact, the level of variation in TF binding sites between closely related species of yeast is substantially larger than that of their regulated orthologs [15]. Adaptive arguments for binding site gain and loss across organisms are based not only on pathway dynamics, but also on the topological (static) properties of the pathway. For instance, properties such as robustness, modularity, redundancy, complexity, and evolvability can be measured by pathway connectivity and have been argued to convey adaptive value to the individual [4,16-18]. A recent study invoked fluctuating phenotypic selection to explain the evolution of scale-free distributions and pathway complexity [19]. However, whether adaptation can operate uniformly on the genome scale to have consistent effect on regulatory complexity is an open debate [20,21]. Assuming regulatory complexity confers fitness, adaptation may select for more complex pathways, hence resulting in the accumulation of binding sites [8]. Analyses under neutral evolutionary scenarios, on the other hand, have argued for the spontaneous gain and loss of binding sites [17].

We analyzed binding sites data from five model organisms: *Escherichia coli K12*, *Saccharomyces cerevisiae*, *Caenorhabditis elegans*, *Drosophila melanogaster*, *Arabidopsis thaliana*. We found a positive correlation between the amount of ncDNA in the organism’s genome and the number of transcription factor binding sites. As most ncDNA is expected to not be under selection, we hypothesize that the accumulation of binding sites, which may functionally affect regulatory systems in terms of multiplicity, redundancy, and degree, can be explained by non-adaptive processes, without invoking selection. To test this hypothesis, we performed realistic population genetic simulation studies using a sequence-based binding site genotype model. Our simulation results indicate that the balance between binding site gain and loss rates, which is largely determined by the amount of ncDNA and the mutation rate, explains the positive correlation in the data. These results confirm our hypothesis. We also found that selection on pathway function, which is determined by its regulatory interactions, does not impose sufficient constraint on the accumulation of binding sites. Further, selection directly opposed to the accumulation of binding sites elicits marginal effect over the mutational bias. We believe that these results have significant implications on the type of information that will be discovered in genome-wide binding site identification studies and on our understanding of how regulatory function evolves and is maintained in non-coding regions.

## Results

### TFBS accumulation positively correlates with the amount of ncDNA

We analyzed binding sites upstream of identified transcription units in five diverse model organisms to investigate the correlation between the length of upstream intergenic regions and the accumulation of binding sites. We curated data from four data warehouses for *E. coli K12*[22], *S. cerevisiae*[23], *C. elegans*[24], *D. melanogaster*[25], and *A. thaliana*[26], representing binding site data for at least 28 TFs per species. We focused only on binding sites residing in the upstream intergenic region and 5’ untranslated region (UTR); downstream or intronic binding sites with putative effect were not counted in this study. In *E. coli K12*, we treated each operon as an independent transcription unit (TU), whereas in the eukaryotic genomes we treated each set of overlapping genes as a single TU. When possible, we used experimentally verified, rather than computationally predicted, binding sites. All binding site data for *D. melanogaster* and *C. elegans* contain only experimentally verified sites, *E. coli K12* and *S. cerevisiae* contain both experimentally verified and predicted sites, and *A. thaliana* is comprised largely of predicted binding sites. In this regard, we minimize the potential effect of false positives in large ncDNA regions by curating mostly experimentally verified sites. We include *A. thaliana* for enhancing the completeness of the study. We matched binding sites with their closest downstream target, similar to [15], and recorded the length of upstream intergenic DNA region as the amount of regulatory substrate available to the particular TU. In the case where forward and reverse strand genes share the same upstream region, binding sites were matched to both genes.

**Figure 1:**
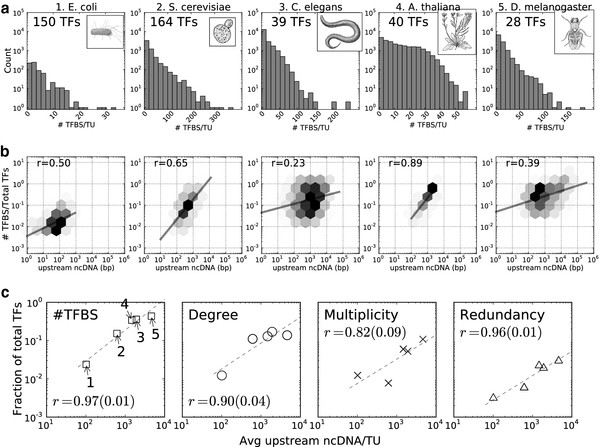
**Regulatory complexity correlates with amount of ncDNA. a.** The histogram of number of transcription factor binding sites (#TFBS) per transcription unit (TU) is given for each organism. The number of TFs represented in the results are given underneath the picture of the organism. **b.** A density map between the amount of upstream ncDNA per TU and the number of TFBS harbored within that region as a fraction of total reporting TFs is plotted for each organism. The provided correlation (*r* equation on plot) is the Pearson correlation coefficient, all with high (*p*<.001) significance. **c.** The regulatory properties for five model organisms is calculated as a fraction of total TFs per species. In the first panel, the each scatter point is labeled for the number provided each organism in **a**; because the average ncDNA length does not change, organisms follow the same order in subsequent panels. Each regulatory property does not necessarily scale between 0 to 1, as only ‘Degree’ is normalized over total TFs per species. A log linear regression is calculated and displayed with the Pearson correlation coefficient and p-value in parentheses.

Across an entire genome, binding site accumulation correlates with the size of the intergenic region, although this correlation may be as low as 0.23 and as high as 0.89 (see Figure 1b). Because binding site formation is subject to many diverse, heterogeneous factors (e.g. binding site length, TF promiscuity, conservation) which vary between species, we do not expect this correlation to be high within the genome of an organism or consistent across organisms; however, we note that the general trend — longer ncDNA upstream regions tend to harbor more binding sites — is indeed supported by empirical evidence. Despite the lower number of TFs sampled in *C. elegans* and *D. melanogaster* in comparison to the other organisms, the distribution of TFBS count per TU matches that of *S. cerevisiae* and *E. coli K12*, following a scale-free pattern (see Figure 1a). The frequency of intergenic regions harboring a given amount of binding sites log linearly decreases as the number of binding sites increase. We observe this pattern regardless of the number of transcription factors, the number of intergenic regions, or the length of the intergenic regions.

The data repository for binding sites in *A. thaliana* constrained upstream intergenic regions to be at most 3,000 nt, although larger upstream regions exist in the genome. We suspect that this truncation of intergenic regions in the *A. thaliana* data repository skews the distribution of TFBS count/TU from that of the other organisms and improves the correlation between TFBS count and ncDNA length (because ncDNA close to transcription start sites have higher TFBS density; Additional file 1). In addition, because the binding site data for the other organisms is largely experimentally verified, we suspect that the strong correlation in *A. thaliana* may be due to a systematic false positive rate in the identification of its binding sites.

### Regulatory complexity positively correlates with the amount of ncDNA

As the amount of ncDNA correlates with the accumulation of TFBSs, and that latter governs the emergence of regulatory interactions, a question naturally arises as to the complexity patterns that emerge in regulatory networks as a function of the amount of ncDNA. To investigate this question, we considered four properties of regulatory pathways: for each TU, the average number of upstream binding sites (we refer to it as ‘#TFBS’), the average number of binding sites per TF that regulates the TU (we call it ‘multiplicity’), the number of unique TFs that regulate the TU (we call it ‘degree’), and the fraction of TUs with more than one regulating TF (we call it ‘redundancy’). To account for the varying amount of TFs for each organism, each reported property was scaled according to the contribution of each TF (by dividing by the number of TFs per organism in this study). We present these values as a function of upstream ncDNA in Figure 1c, and find strong positive correlations between the amount of ncDNA upstream of TUs and the regulatory properties under study (shown in Figure 1c). Although these properties are derived from binding site accumulation, their values are not completely determined by the number of binding sites. For instance, binding sites accumulation may increase degree without affecting multiplicity. Similarly, if unique binding sites accumulate, then multiplicity may increase without affecting degree.

As the amount of ncDNA per TU increases, an *arbitrary* TF would increase TFBS and multiplicity by binding to more sites upstream of a gene, degree by interacting with more genes, and redundancy by redundantly regulating more genes.

These results provide strong empirical evidence to the rising regulatory complexity that accompanies increasing amounts of regulatory substrate, and combined with the strong correlation presented in Figure 1c, give rise the question: Is the accumulation of binding sites driven by adaptive forces acting upon the regulatory pathways or neutral forces deriving from the amount of ncDNA? To answer this question we investigate, through simulations, the effect of ncDNA on the accumulation of binding sites and determine its relative strength against recombination, selection on pathway function, and selection on pathway topology. Through these experiments we measure the effect of non-adaptive versus adaptive forces on the accumulation of binding sites and quantify evolutionary advantages and disadvantages of environments of high binding-site gain and high binding-site loss genomic by the availability of innovative phenotypes (evolvability).

### Simulations reveal the role of drift and ncDNA on TFBS accumulation

In [17], Lynch defined parameter *α*=_*ul*_/_*ug*_, where _*ul*_ is the rate of binding site loss and _*ug*_ is the rate of binding site gain. When *α*≪1, binding sites are much more likely to be gained than lost, and when *α*≫1, binding sites are much more likely to be lost than gained. As Lynch showed, this mutational bias (*α*) can be parameterized via two empirical sequence properties: *α*=^4*n*^/*L*, where *n* is the average length of a binding site and *L* is the length of DNA which may harbor a binding site. This parameter resolves from _*ul*_=*un*and $u_{g}=\mathrm{unL}/4^{n}$, where *u* is the mutation rate per base pair (for a derivation of these equations see [17]). Since the length of a binding site varies little in comparison to the amount of ncDNA in genomes, *α* is largely a function of *L*, the amount of regulatory substrate flanking a regulatory target. Further, since the amount of ncDNA increases with genome size, *L* scales empirically over several orders of magnitude across the tree of life: prokaryotes have *α*=1^04^ and mammals and land plants have *α*=1^0−3^.

The regulatory machinery and binding site length differ greatly between the species in this study. As such, we assume a very simple model of regulation shared by all organisms which incorporates only TFs and their binding sites. The sites to which a TF binds can be characterized with a binding site motif. In this simple regulatory model, we assume binding site motifs match exactly one sequence of length *n*, but in practice most binding site motifs match several sequences; therefore, we cannot use the length of known binding site motifs to accurately parameterize *n*. Instead, we are interested in parameterizing *n* with a value somewhere between the number of sites which must match exactly (consensus sites) and the total length of the binding site motif. To account for this, we calculated the average number of consensus sites and binding site motif length for *E. coli K12* and the eukaryotes in the study (Additional file 1). We found that reasonable values of *n* for *E. coli K12* are in the range 10-20 bp and in the range 5-10 bp for eukaryotes.

We simulated the evolution of a 5-gene pathway, with each gene as its own TU, in a population of size *N* for 1^09^ generations across values of ncDNA (*L*) ranging from 1^02^to 1^04^ and binding site size (*n*) ranging from 10−20 (prokaryotes) and 6−10 (eukaryotes), under models of binding site mutation (gain/loss) and genetic drift.

**Figure 2:**
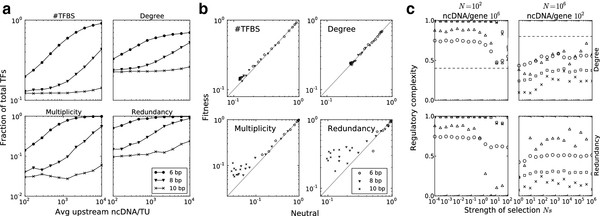
**Explaining regulatory complexity with neutral evolution. a.** Simulations using a 5-gene pathway under mutation and drift yield log-linear increases in the number of binding sites (#TFBS), degree, multiplicity, and redundancy with respect to the amount of ncDNA per transcription unit. Each line corresponds to a different binding site length, given in the legend. **b.** The results from running population genetic simulations on a random fitness landscape (y-axis) compared with the results from neutral - mutation and drift - simulations (x-axis). Deviations from neutrality are points off the diagonal. **c.** The pathway properties respond to the contributing factor *s*, which goes from insignificant (*s*=1^0−6^) to dominant (*s*=1). The x-axis denotes the strength of selection in a population, or *Ns*. The rows denote the property under selection and the columns represent the high gain and high loss environments, respectively. The optimal value was chosen against the neutral bias and is denoted by a dashed line for degree. In the case of redundancy, the optimal value was 0 (high gain) and 1 (high loss). Shapes denote the regulatory complexity measurements of #TFBS (□), degree (○), multiplicity (×), and redundancy (△).

We set the population size *N* as a function of *L*, based on the empirical correlation between genome size and population size—as genomes increase in length, populations tend to shrink—such that *N*=1^02^^(*uL*)−1^, where the base pair mutation rate *u* remained constant at 1^0−9^for all simulations [27,28]. For each *N*, amount of ncDNA *L*, and binding site length *n*, results were averaged over 80 replicate stochastic simulations. The results (Figure 2a) show that the four properties under investigation log-linearly increase with respect to the amount of ncDNA, following the pattern found in the empirical data. The binding site length has an effect on the slope and position of this increase: shorter binding sites spontaneously arise more frequently than longer ones, leading to higher values of binding site accumulation, degree, multiplicity and redundancy. The binding site length (8 bp) that elicits a similar pattern to the empirical data is the length between the average number of consensus sites (6 bp) and the length (10 bp) for eukaryotes. In comparison, a binding site length of 10 bp shows minimal response to increasing amounts of ncDNA and a length of 6 bp ‘saturates’ the degree, multiplicity, and redundancy values of the simulated pathway. For *E. coli K12*, we performed further simulations to understand the effect of increasing the binding site size to 20 bp when *L*≈100. Under these simulations, we found a minimal response in regulatory complexity to binding site lengths beyond 10 bp (Additional file 1). Because we implement a viability constraint that all genes must remain regulated, the effect of increasing the binding site length, at some point, is masked by the inability of pathways to lose any more binding sites. We find that this point, under our simulation settings, is around 10 bp for short upstream ncDNA regions.

In [17] it was shown that redundancy increases with increasing amounts of ncDNA; however, our results show that this is a result of increasing binding site accumulation which also drives degree and redundancy. Large amounts of ncDNA in the genome result in higher complexity of the regulatory pathways in the population. Thus, the log-linear increase of the binding site accumulation previously identified in the empirical data is explainable by mutation and genetic drift.

To investigate whether recombination, another neutral evolutionary force, plays any role in the observed correlations, we simulated recombination using two methods, namely between genes and between binding sites, and found that neither had strong effect on the accumulation of TFBS (Additional file 1). Applying even strong levels of recombination between binding sites did not elicit change in binding site accumulation. Previous work identified the feasibility of recombination to cause redundancy in small regulatory networks over long evolutionary time scales [17]. When applied to binding site accumulation, however, recombination between binding sites yields minor effect over that which is determined by mutation and drift.

### Selection and TFBS accumulation

While simulation results thus far confirm an essential role of neutral evolutionary forces in shaping TFBS accumulation, they do not rule our the role of adaptive forces. To investigate whether selection aids in explaining the observed trends of TFBS accumulation, we modeled fitness in two ways: acting on the regulatory pathway function via an *abstract phenotype* fitness function (see Methods), and acting on the regulatory pathway complexity via *pathway property* fitness functions properties using properties defined above.

In employing the abstract phenotype fitness function, an initially monomorphic population evolves on a soft fitness – meaning the fitness function is not binary – landscape where each phenotype class is assigned a random selection coefficient. A phenotype class is defined as a vector of the ‘up’ or ‘down’ regulation of each gene in steady state [16] (see Methods). The population evolves on this landscape for 1^09^generations. The results from these simulations are given in Figure 2b.

Although the fitness is based on the pathway steady state dynamics, there are no significant deviations from the patterns found in non-adaptive simulations for the number of binding sites and degree across all binding site and upstream ncDNA sizes. For a 10 bp binding size length, selection on steady state dynamics tended to increase the frequency of multiplicity and redundancy in the pathway across all sizes of ncDNA over the results found by neutral simulations. In sparse pathways, loss of a binding site often leads to loss of function — so selecting for a certain steady state dynamics would enhance this effect over the viability constraint imposed in the neutral simulations. Thus, in sparse networks with long binding sites, one would expect to find signal for conserved binding sites. On the other side of the spectrum, when networks are not sparse, and the spontaneous gain rate of binding sites is relatively equivalent to their loss rate, selecting on a specific pathway function does not affect regulatory complexity. Therefore, constraining a population to evolve within certain pathway dynamics restrictions (as imposed by the fitness landscape) has minimal effect on binding site accumulation beyond the neutral bias, but may have an affect on multiplicity and redundancy in prokaryotic genomes. So, the general application of selection on pathway dynamics does not seem to affect binding site accumulation on the genome scale.

If selection acted not entirely on pathway function, but instead to some extent on complexity itself, then we can model such a relationship with a selection coefficient directly on a topological pathway property associated with complexity. In this experiment, the fitness of a pathway genotype is a function of its redundancy and average degree. Here, we ran simulations on both ends of the ncDNA amount spectrum (ncDNA amount =100 and ncDNA amount =1^06^) to understand the effect of selection operating directly on pathway properties, using the *pathway properties* fitness model. Unlike the biologically realistic rates and population sizes used in the previous sections, *s*—the contribution factor of the complexity property—cannot be readily estimated from biological evidence. So, in these experiments, we performed a logarithmic parameter sweep of *s* from insignificant (*s*=1^0−6^) to dominant (*s*=1). The results of this experiment are shown in Figure 2c.

The strength of selection is relative to the population size, where the magnitude of genetic drift overpowers selection when *s*<1/*N* or *Ns*<1. In Figure 2c, there is a noticeable phase shift when *Ns*>1 in the left column (ncDNA=1^06^and *N*=100). For the right column (ncDNA=1^02^ and *N*=1^06^), the minimum value of *s* is 1^0−6^, which is equivalent to 1/*N*, so selection overpowers genetic drift.

Unlike the experiments above, where fitness had no significant effect on pathway properties, direct selection on pathway properties can force deviations from the pattern caused by mutation and drift. However, selection on average degree effects the other complexity properties differently than selection on redundancy. When average degree is under selection, all other properties respond in accordance by covarying with the change in average degree. On the other hand, when redundancy is under selection, the other properties retain the neutral bias.

These experiments demonstrate that specific pathway properties can be driven by adaptive forces when selection operates as a function of that property, and that the signature of selection on primary or derived complexity properties are different. We may expect that if selection were to operate on complexity, it would be on a derived property, like redundancy or multiplicity.

### ncDNA increases innovation

**Figure 3:**
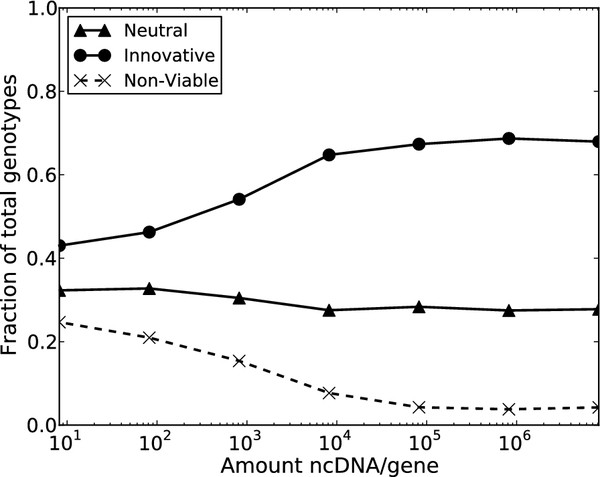
**Amount of ncDNA affects innovation and robustness.** The fraction of genotypes visited by evolving populations with respect to ncDNA/gene identified the positive effect of regulatory substrate length on the availability of innovative phenotypes.

Although increasing complexity may be a byproduct of neutral mechanisms acting on ncDNA, large regions of ncDNA may serve a beneficial role in evolution. Specifi- cally, we investigated the ‘evolvability’ of a population as a function of its ncDNA by comparing the proportion of novel phenotypes discovered during the course of evolution. The accessibility of phenotypes in the evolutionary landscape is an informative property explaining the ability of populations to adapt to novel environments [29-32]. We parameterize the accessibility of phenotypes to a population by categorizing visited phenotypes during the course of evolution as: neutral (same as initial phenotype), innovative (different from initial phenotype), or non-viable. We simulated replicate populations across a spectrum of ncDNA lengths; the results are displayed in Figure 3.

Under the model employed in our simulations, the topology of a pathway and its steady-state dynamics are not dependent on the amount of ncDNA; rather, the amount of ncDNA only modifies the manner in which populations explore the space by imposing a mutational bias. Pathways with different amounts of regulatory substrate may tend to either gain or lose binding sites, which in turn affects the accessibility of steady-state phenotypes. Intuitively, binding site loss would dismantle pathways more often than their gain. And so, populations of pathways that tend to gain binding sites over losing them — that is to say, populations with genomes that harbor large ncDNA regions — would have access to more innovative phenotypes over populations with shorter ncDNA regions. In this manner, we can positively correlate ncDNA with phenotypic innovation and organism evolvability.

## Discussion

By analyzing data from five model organisms, we found that the mutational bias imposed by the amount ncDNA, in addition to population size, fosters or inhibits the accumulation of binding sites neutrally. Further, purifying selection on pathway function or on regulatory complexity itself shows minor effect over the mutational bias. Our results do not diminish the role of selection in conserving functional binding sites; instead, they demonstrate that selection must operate within the bounds of complexity determined by the amount of regulatory substrate, or ncDNA. These results are corroborated by convincing empirical evidence—the strong correlation between ncDNA and binding site accumulation—from a diverse group of model organisms.

To validate our hypotheses, we performed realistic population genetic simulations which were parameterized with accurate and empirically-calculated rates. We used a model of pathway genotype that encodes binding sites instead of regulatory interactions [16,17,32], which we believe is more biologically accurate and leads to different results. For instance, our binding site based model demonstrated that recombination between binding sites is a minor force compared to the mutation bias in developing redundancy; this result is considerably different than what was found previously under an interaction-based model [17]. Our results are supported by the empirical data which shows that the levels of redundancy in *E. coli K12* seem to agree with the log-linear trend against ncDNA. Furthermore, our results depict the significant effect binding site length can have on their accumulation when the length of ncDNA is large: an expansion of 2 bp in length (from 8 bp to 10 bp) can decrease the number of binding sites, degree, multiplicity, and redundancy by almost an order of magnitude. When there is a small amount of ncDNA, regulatory complexity is less affected by binding site length. This observation is especially interesting because binding sites are known to be longer in prokaryotes (10-20 bp) than in eukaryotes (6-10 bp). Due to this large difference in length, the short binding sites in eukaryotes, working in concert with long stretches of ncDNA, may play a significant role in the emergence of regulatory complexity which characterizes ‘higher organisms.’

While we only modeled de novo binding site gain, binding sites may arise through other evolutionary mechanisms, including duplication by transposons, repeated elements, or non-homologous recombination [1]. We therefore interpret our results as a baseline of the potential accumulation of binding sites, and recognize that binding site accumulation in reality could be increased by these evolutionary mechanisms. Since these mechanisms may actually extend ncDNA regions, their incorporation in future work may provide further insight into the fundamental ties between ncDNA and regulatory complexity. Under the time scales simulated in this study, ncDNA regions are known to contract and expand, which would have a significant effect on the accumulation of binding sites and regulatory complexity. Further, the amount of ncDNA may be under selection, as a result of adaptation on genome size [33,34]. Regardless of the evolutionary origin of the regulatory substrate, our results still explain a non-adaptive increase in regulatory complexity through mutation and genetic drift. In fact, we showed that large ncDNA regions potentially increase the evolvability of a population which, in turn, may confer an adaptive value to the population. However, according to our simulations, this adaptive value is a byproduct of neutral evolution and not necessary for explaining empirical patterns in regulatory complexity.

### A genotype-phenotype space perspective

Our simulation results are based on a certain pathway dynamics and mutation model; however, we believe that our findings generalize, which we can better understand on a genotype-phenotype space. In this abstract space, genotypes are immediate neighbors if they can be transformed into one another by a single mutation event [35]. Neighboring genotypes with the same phenotype form a connected sub-space—the *neutral space*—wherein mutations occur ‘invisibly’ to selection. Over the course of evolution, a population moves in this space by acquiring neighboring genotypes of existing ones through mutation or recombination. By selecting a certain pathway genotype and phenotype model, we implicitly create a genotype-phenotype space.

Neutral spaces for the regulatory pathway genotype are predicted to be far-reaching in terms of mutational distance, and so a pathway may undergo substantial mutation while still maintaining the same phenotype [16]. In this case, a population may accrue binding sites according to the mutational bias, implementing the same pathway function with either many or few binding sites. However, in the case of disjoint neutral spaces, there are many known evolutionary mechanisms to overcome these ‘fitness valleys,’ one of which is relying on genetic drift to move the population across it [36,37]. This is a reasonable mechanism to support the increasing regulatory complexity in ‘higher’ organisms with small population size.

Let us consider two populations in an identical genotype-phenotype space: one with large amounts of ncDNA per gene and small population size (e.g., the human population), and the other with small promoters and large population size (e.g., a bacterial population). Because of the mutation bias pushing it towards or away from binding site accumulation, each population will evolve differently on the exact same space. If regulatory complexity and innovation co-occur with binding site accumulation, which we argue to be the case, then a population may develop these properties regardless of adaptation. Over long evolutionary time scales, and assuming very little of the fitness landscape, the large genome population will develop regulatory complexity and ‘innovative’ pathways because it is biased towards binding site gain. On the other end of the spectrum, due to a binding site loss bias, the bacteria-like population will neutrally inhibit complexity and generate more non-viable mutants.

### Implications

If the accumulation of binding sites follows neutral trends, then most binding sites may actually be neutral to function, and therefore encode very little ‘meaningful’ information in terms of understanding the transcriptome. Consequently, the identification of binding sites would yield minimal functional insight and actually mining functional signal from their identification would be muddied by over-complicated regulatory interactions. Ultimately, the *cis*-regulome is not a clean functional network, but instead a ‘messy’ data source filled with the effects of non-adaptive forces.

According to our work, the expansion of ncDNA would neutrally give rise to more complex networks by way of binding site accumulation. Thus, if we integrate these results with Lynch and Conery’s hypothesis on the origin of genome complexity [38], organismal complexity is more a result of decreasing population size than adaptation. Lynch and Conery hypothesized that expanding, maladaptive non-coding regions resulted from the weakening of selective pressure due to significant decreases in the effective population size [38]. The amount of regulatory substrate, or ncDNA, is then a function of population size; and so, if binding site accumulation, which leads to regulatory complexity and organismal complexity, is a function of ncDNA, then it is also a function of population size. Integration with our results would explain organismal complexity as the eventual evolutionary result of decreasing population size. Our results provide an important explanatory step between the evolution of genome complexity and regulatory complexity using binding site accumulation.

A revised, neutral understanding of how regulatory complexity evolves has a dramatic impact on many directions in microbiology and genetics. The spontaneous gain rate of binding sites in ‘higher organisms’ would lead to unknown polymorphisms in the regulatory regions of genes, between individuals, and even between cells in a body, which is a significant complicating factor to treating non-Mendelian diseases like cancer. These results transfer to synthetic biology, where design principles of regulatory circuits must accommodate the neutral evolutionary forces that will wreak havoc on ‘industrial’ (wild) populations. Only pathways that can withstand the neutral bias of their host population will remain as designed; pathways that do not have adequate evolutionary ‘supports’ in place may either spontaneously decompose or augment, depending on the amount of ncDNA and population size.

## Conclusions

The recent availability of genome-wide transcriptome data across several model organisms allows for the analysis of transcription factor binding site accumulation. These binding sites form regulatory networks which determine the dynamics of many critical developmental and cellular processes. Consequently, recent research efforts have pushed towards the identification of high confidence binding sites across the entire genome. The success of these studies, therefore, depends on the fact that binding sites encode functional information on the genome scale; however, the extent to which this assumption is true has yet to be tested. In this work, we show that neutral evolutionary forces alone can explain binding site accumulation, and that selection on the regulatory network function does not alter this finding. If neutral forces drive the accumulation of binding sites, then, despite selective constraints, organisms with large amounts of ncDNA would evolve functional, yet ‘over-complicated’, networks. Organisms with streamlined genomes – containing small amounts of ncDNA – tend to evolve similarly streamlined pathways. However, we also show that these complex networks may provide important regulatory substrate for innovation, improving the ‘evolvability’ of the organism. Ultimately, The *cis*-regulome is not a clean functional network crafted by adaptive forces alone, but instead a data source filled with the noise of non-adaptive forces. From a regulatory perspective, this evolutionary noise manifests as complexity on both the binding site and pathway level, which has significant impact on how we view, sample, create, and, ultimately, understand these important, yet enigmatic, systems.

## Methods

### Fitness model

Fitness is modeled under three scenarios: 

1. *viability* - requires that all genes in the pathway are regulated, given by (*g*), 0 otherwise.

2. *abstract phenotype* - each phenotype class (out of ^2*k*^possible classes) are assigned a random fitness value from a uniform distribution. The fitness of a genotype is assigned based on these fitness-to-phenotype class mappings. Each simulation regenerates a new fitness-to-phenotype mapping.

3. *pathway properties* - for a given pathway genotype *g*,

$${\text{fitness}}_{\mathrm{prop}}(g)=\text{viability}(g)|1.0-s(L_{\mathrm{prop}}-\mathrm{prop}(g))|,$$

where 0≤_*Lprop*_,*prop*(*g*),*s*≤1. The variable *s* is a scaling factor which represents the strength of selection contributed by deviation from the optimal property _*Lprop*_. This contributing factor is modeled as *s*, which may be insignificant (*s*=1^0−6^) to substantial (*s*=1).

### Population simulations

Population genetic simulations were used to understand the combined effect of mutation rate, recombination rate, population size, and fitness on pathway evolution. In these experiments, we simulate a population of regulatory pathways over *G* generations, where each pathway is comprised of genes (or TUs) and their upstream ncDNA divided into regions which may harbor binding sites. No sequence data is simulated; rather, we simulate genotypes on the pathway level. In this study, we simulate a 5-gene pathway where the upstream ncDNA for each gene may harbor up to 10 binding sites. We assume the population to be haploid, panmictic, and constant in size.

Given a population size *N*, generation time *G*, mutation rates _*ug*_ and _*ul*_, inter-binding site recombination _*rb*_, and inter-gene recombination _*rg*_, the population simulations performed in this study used the following method. First, a pathway genotype is randomly generated, such that *cM* binding sites are occupied, where *M* is the maximum number of binding sites and *c* is a proportion (*c*=0.5 in all experiments). The population of size *N* is then evolved for *G* generations. Results from a simulation are weighted averages of allele properties based on their frequency. Alleles are sampled after a burn in period of *G*/2, which allows for the population to reach steady state.

For simulations incorporating fitness or phenotype calculations, we append the aforementioned process with additional steps. The phenotype of a pathway is modeled as the steady-state concentrations of gene productions, which we calculate using a simple discrete method [16]. This method requires a *k*-binary input signal, where *k* is the number of genes in the pathway. This input signal provides the starting concentrations for gene products in the pathway.

Please refer to the Additional file 1 for further details.

## Authors contributions

Both TR and LN contributed equally to this work.

## Supplementary Material

Additional file 1 Supplementary Information. Supplementary information contains further data curation, analyses, and simulation details. Click here for file
