# Supplementary material for: ncDNA and drift drive binding site accumulation
Source: BMC Evol Biol. 2012 Aug 30;12:159. doi: 10.1186/1471-2148-12-159 (PMC3556125; doi:10.1186/1471-2148-12-159)
Supplement: Additional file 1 — Supplementary Information. Supplementary information contains further data curation, analyses, and simulation details. [file 1471-2148-12-159-S1.pdf]

# Supporting Information
# ncDNA and drift drive binding site accumulation

Troy Ruths & Luay Nakhleh

## Contents

**1 Results** **3**

1.1 Distribution of TFBS . . . 3

1.2 Distribution of TFBS length . . . 3

1.3 Effect of recombination on TFBS accumulation . . . 4

1.4 Effect of binding site length on TFBS accumulation in *E. coli K12* . . . 6

1.5 Why recombination in short promoters does not lead to redundancy . . . 6

**2 Encoding regulatory pathways** **9**

2.1 Genotype . . . 9

2.2 Phenotype . . . 11

2.2.1 Discrete . . . 11

2.2.2 Viability . . . 12

2.3 Fitness . . . 12

**3 Evolving pathways** **14**

3.1 Mutation . . . 14

3.2 Recombination . . . 16

3.3 Population simulations . . . 16

**4 Data provenance** **17**

4.1 *E. coli* . . . . . . . . . . . . . . . . . . . . . . . . . . . . . . . . . 17

4.2 *C. elegans* and *D. melanogaster* . . . . . . . . . . . . . . . . . . . . . 17

4.3 *S. cerevisiae* . . . . . . . . . . . . . . . . . . . . . . . . . . . . . . . 18

4.4 *A. thaliana* . . . . . . . . . . . . . . . . . . . . . . . . . . . . . . . . 18

4.5 On the quantity of data . . . . . . . . . . . . . . . . . . . . . . . . . . 18

# 1 Results

## 1.1 Distribution of TFBS

We calculated the distribution of binding sites as a function of distance from the TSS. For a sliding window, the TFBS density was calculated as the number of TFBS within the window normalized by: (1) the window size, (2) number of promoters which at least the length of the extent of the window, and (3) the total number of TFs. The results are presented in Fig 1. The distribution of TFBS density is non-uniform for all organisms, following an exponential distribution: binding sites are more likely to occur closer to the TSS, which is corroborated by previous work [1]. TFBS density does not diminish to 0, but rather plateaus around $5 \times 10^{-5}$ #TFBS/nt.

In addition, the distribution of binding sites per TF is heterogeneous. We calculated the number of binding sites in a given window upstream of the gene for each TF and presented the results in Fig 1.

## 1.2 Distribution of TFBS length

The length of binding sites varies between both prokaryotes and eukaryotes and transcription factors. To better understand this distribution and improve the parameterization of our simulations, we downloaded the distribution of binding sites for *E. coli K12* from RegulonDB and several eukaryotes (*D. melanogaster*, *C. elegans*, and *S. cerevisiae*) from JASPAR (shown in Fig 2) [2]. Because we parameterize our simulations with the number of sites that have to match exactly, we are interested in determining the 'effective' binding site length which is somewhere between the number of consensus sites and the total length of the binding site motif. We calculate the average consensus sites and binding site length for *E. coli K12* and select eukaryotes in this study and find the effective binding site to exist between 10-20 bp for *E. coli K12* and 5-10 bp for eukaryotes.

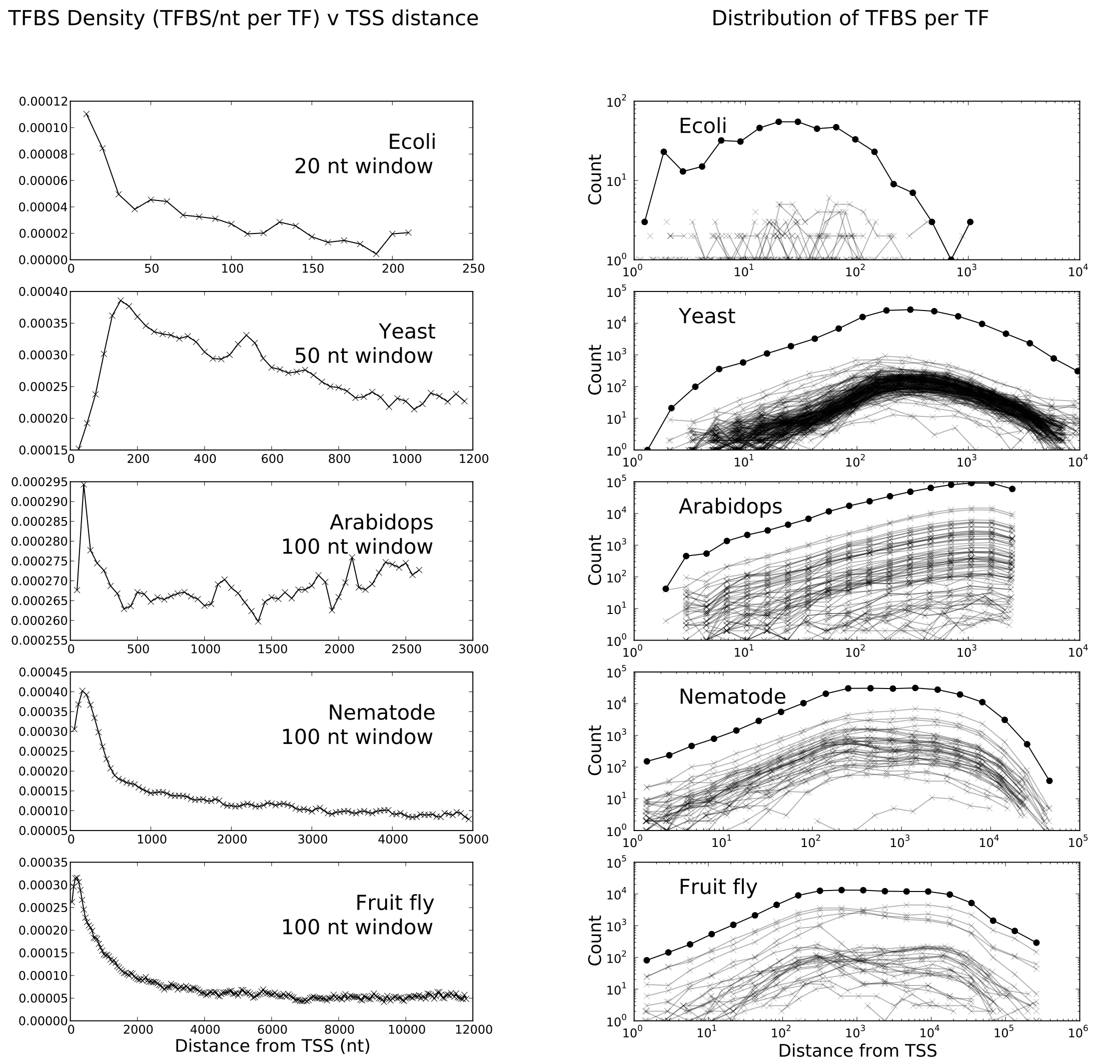


**Figure 1. Left**. The TFBS density (#TFBS/nt) was calculated for a sliding window along upstream ncDNA regions of TUs. TFBS density is normalized by the number of TUs and TFs in the study. **Right**. The distribution of TFBS broken out by TF is plotted for each organism. The distribution of all TFs is denoted by a solid black line.

## 1.3 Effect of recombination on TFBS accumulation

We simulated the effect of recombination on TFBS accumulation on bacteria-like populations with an amount of ncDNA $\approx$ 100 bp in length and a population size of $10^6$ individuals. Simulations lasted $10^6$ generations. We only simulated organisms with small

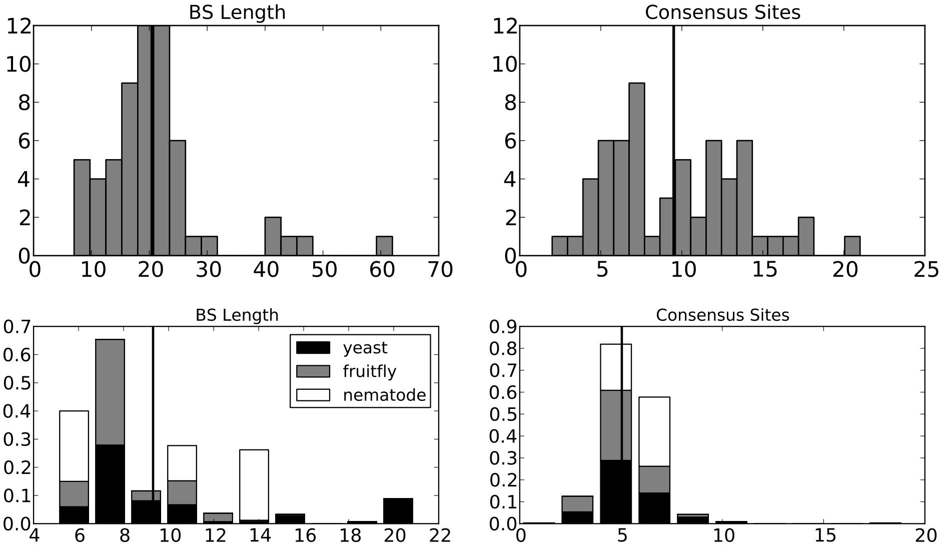


**Figure 2.** The distribution of binding site width (right) and consensus sites (right) for *E. coli K12* (top) and several eukaryotes (bottom). The solid vertical lines display the mean length depicted in each plot.

regulatory regions because in previous work recombination between binding sites was argued to promote redundancy in this type of population - one that did not have a mutational bias towards redundancy [3].

The recombination rate between two locations can be calculated from the nucleotide distance $d$ and the recombination frequency per base pair $c$ as $r \approx 0.5(1 - e^{2dc})$. When $d$ is small, $r \approx dc$. The recombination rates for prokaryotes are on par with the per base pair mutation rates ($c/u \approx 1$) [3]. Therefore a realistic recombination rate inside regulatory regions for prokaryotes would be $r = dc = 10^1 \times 10^{-9} = 10^{-8}$. We performed a parameter scan across both types of recombination - inside regulatory regions and between regulatory regions - and present the results in Fig 3. Both recombination between regulatory regions and inside regulatory regions elicit a minor effect on binding site accumulation. Even simulated recombination rates which exceed realistic values produce minimal change to binding site accumulation. Further, recombination between binding sites seemed to decrease redundancy in the simulated population.

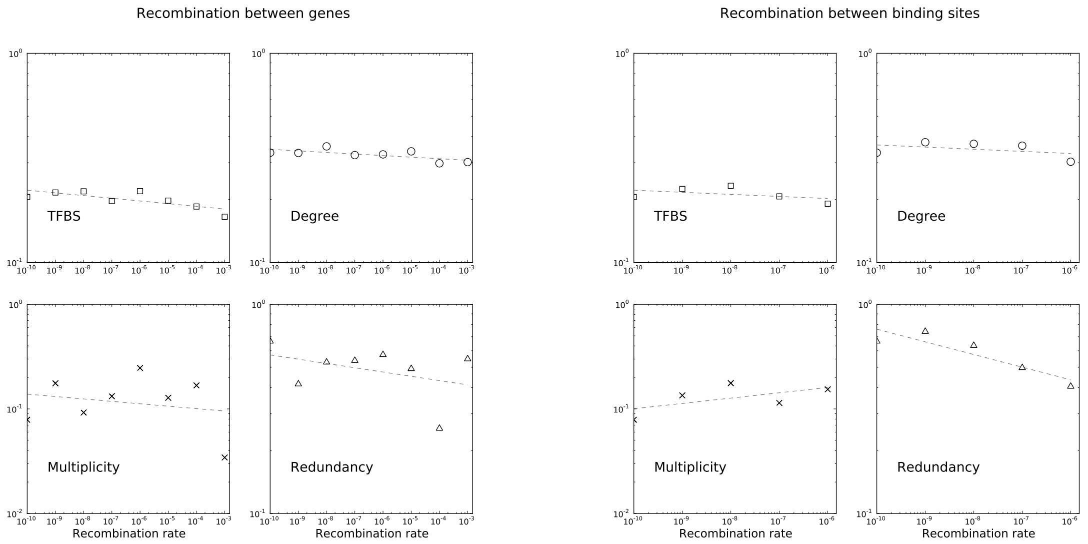


**Figure 3.** Simulations incorporating recombination between and inside regulatory regions have minor effect on binding site accumulation.

## 1.4 Effect of binding site length on TFBS accumulation in *E. coli K12*

We simulated long binding site sizes (10-20 bp) under *E. coli K12* simulation settings ($N = 1e9$, $L = 100$) to understand the effect of binding site size on TFBS accumulation (see Fig 4). We found no response in number of TFBS and degree for binding sites longer than 10 bp. Redundancy and multiplicity continued to decrease in binding sites longer than 10 bp, but not to the same scale as what occurred between 6-10 bp. We attribute these results to the viability constraint which requires genes to remain regulated and so limits the binding sites available to be lost. This reduces the effect of the longer binding sites, which are easier to lose and harder to gain.

## 1.5 Why recombination in short promoters does not lead to redundancy

Previous work presented a theoretical argument that recombination between binding sites in the promoter regions of prokaryotic species can lead to redundancy on the network level [3]. Our results argue otherwise. To understand why this is the case, let us examine

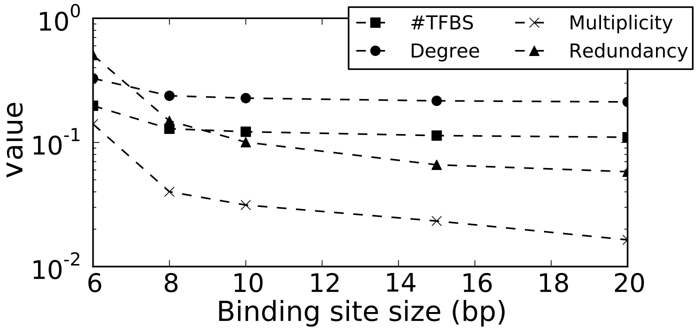


**Figure 4.** Results for simulations with $N = 10^9$ and $L = 100$ for varying binding site sizes.

the possible recombination scenarios within regulatory regions containing binding sites. Consider a stretch of regulatory ncDNA of length $L$ discretized into $g$ sections such that each region harbors at most one binding site of length $n$. When recombination occurs in one of these sections, each parent may have a binding site or only one parent has a binding site. There are many outcomes for such a recombination: for the case where both parents have a binding site, the outcomes are depicted in Figure 5.

First, consider what happens if only one parent has a binding site. Based on where the crossover occurs relative to the binding site, the site may be unchanged if it is upstream, moved if it is downstream, or lost if it is coincident. The probability of each of these events based on the size of this binding site region $L/g$ is shown in Figure 6. The probability that recombination destroys a binding site decreases exponentially with respect to the size of the binding region. As one would expect, it is equally probable for the binding site to be upstream or downstream of the recombination event. But in any of these cases, because neither recombinant gains a binding site through the event, redundancy is not promoted.

In the case where both parents have a binding site, there are several more scenarios to consider. These scenarios are illustrated in Figure 5. For scenarios **C** and **D** there are symmetric cases where the recombinant TFBS are mirrored. For example, **A** and **B** show the symmetric case for binding sites co-occuring upstream or downstream of the crossover. Of interest are the scenarios that cause redundancy (**C**), loss of one binding site (**D**), and loss of both binding sites (**E**, **C**). Figure 6 shows the impact of each scenario with respect

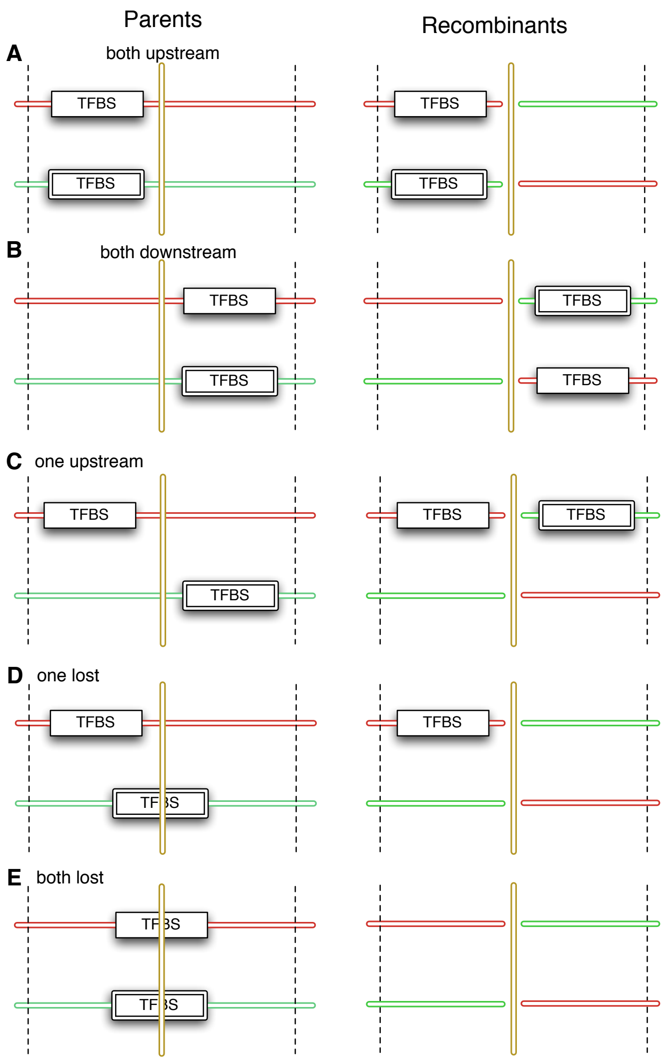


**Figure 5.** All possible results of a recombination event in a critical binding region (one of $g$ in each regulatory region).

to the length of a binding region $L/g$. When $L/g = n$, the crossover is guaranteed to eliminate both TFBS. The probability of each scenario is a function of the binding region size and length of a binding site ($L/g$). Interestingly, for bacteria, $L/g$ is likely less than 100 bp, and so there is a significant probability that recombination would eliminate one or both binding sites (scenarios **C**,**D**,**E**). For eukaryotes, $L/g$ is much larger, and a greater chance exists of swapping binding sites or causing redundancy (**A**,**B** and **C**). However, even in this case, the scenario which promotes redundancy happens only 1/3 of the time. Hence, it is unlikely that recombination would promote redundancy on the network level.

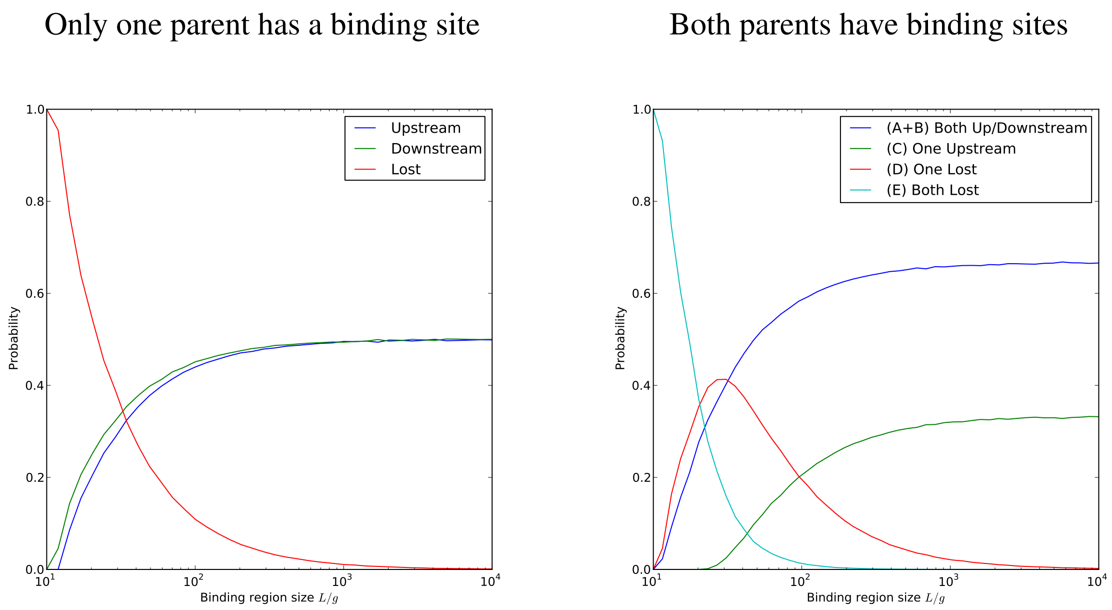


**Figure 6.** The probability of each possible recombinant scenario if only one parent has a binding site (left) or both parents have a binding site (right). The letters in the figure on the right refer to the scenarios mentioned in Figure 5.

In the previous work which argued that recombination may promote redundancy, simulations were run using a network-level model. Since the scenarios we discuss are results of features on DNA sequence, the network level approach would be unable to simulate them.

# 2 Encoding regulatory pathways

## 2.1 Genotype

Regulatory pathways consist of genes that encode transcription factors that either activate or inhibit the transcription of their own or other genes. The binding sites that mediate this regulation exist along the sequence, both upstream, within, and downstream of the gene. To preserve the sequential ordering of binding sites, the promoter and enhancer region for a gene is discretized into $g$ regions, where only one binding site may occur in each of these $g$ regions.

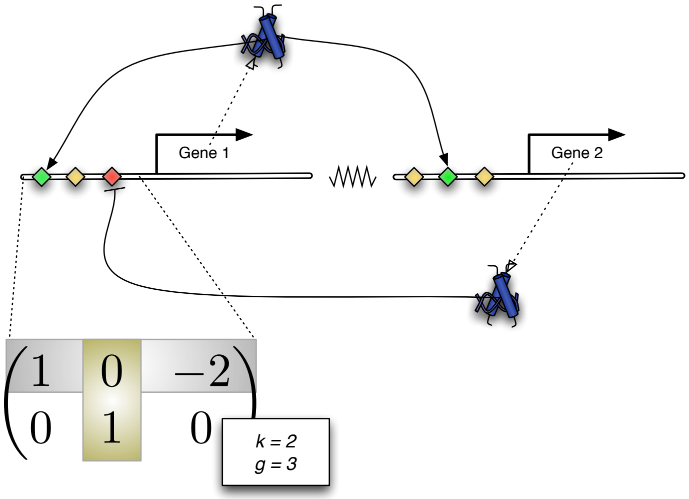


**Figure 7.** A cartoon that illustrates a matrix for a genetic pathway comprising of genes, transcription factors, and binding sites. The matrix representation denotes the binding sites and their affinity. This discretization of binding sites represents where a binding site may arise.

The pathway genotype is econded as a $k \times g$ matrix, where $k$ is the number of genes in the pathway, as shown in Figure 7. An allele is an instantiation of the $k \times g$ matrix:

$$M = (\, b\,)_{i,j}\,,$$

where $b_{i,j}$ encodes the status of the $j^{th}$ binding region for the $i^{th}$ gene. The regulatory region for gene $i$ is encoded as row $i$ in the matrix, and the columns preserve the ordering of binding sites along the sequence. The binding site status, or $b_{i,j}$, is either $0$, for no binding site present, or a value $[1, k]$ for activation and $[-1, -k]$ for inhibition, representing the index of the transcription factor $|b_{i,j}|$ that binds to the given site. Formally, $b_{i,j} \in \{-k, ..., k\}$ and

$$b_{i,j} = \begin{cases} d > 0 & i \text{ activated by } d \\ 0 & \text{no regulation on } i \\ d < 0 & i \text{ inhibited by } d \end{cases}$$

In this work, we use $k = 5$ and $g = 10$.

It is important to stress that this data structure is not an adjacency matrix. Previous approaches used adjacency matrices to encode genetic networks, but this leads to a departure

from the sequential underpinnings of regulatory pathways [3, 4, 5]. An adjacency matrix is a square matrix $A_{i,j}$ ($k \times k$) that encodes the weight, or presence, of an edge between two vertices $i$ and $j$. For a given pathway topology (or adjacency matrix) there are several configurations of binding sites along the genomic sequence. Hence, mutations and recombinations that could greatly change the binding site data structure may appear neutral on the pathway topology.

To calculate the adjacency matrix from a regulatory pathway allele:

$$A_{i,j} = \sum_{0<h\leq g} \delta(|b_{j,h}| - i)sign(b_{j,h}), \tag{1}$$

where

$$\delta(x) = \begin{cases} 1 & x = 0 \\ 0 & \text{otherwise} \end{cases}$$

$$sign(x) = \begin{cases} 1 & x \geq 0 \\ -1 & x < 0 \end{cases}$$

The edge weight is positive for activation and negative for inhibition. If there is an activating and inhibiting binding site for the same gene in a given promoter, the adjacency matrix will report a value of $1 + -1 = 0$, which hides the regulatory effect of the binding sites. More complicated methods for determining the regulatory influence of competing binding affinities can be investigated in future work.

## 2.2 Phenotype

### 2.2.1 Discrete

The work by Wagner determines an expression pattern of $k$ genes as a time series $S(t) = [S_1(t), S_2(t), ..., S_k(t)]$, but calculates $S_i(t+\tau) = \sigma[\sum_{j=1}^{k} w_{ij} S_j(t)]$, where $\tau$ is some constant time step, $\sigma$ is the sign function, and $w_{i,j}$ is the regulatory influence between gene-$i$ and gene-$j$ [4, 5]. A product state at any time is either -1 for down regulation, 0 for no regulation, or 1 for up-regulation. In the update equation $S_i(t+\tau)$, influence outside of these values are scaled using the sign function ($\sigma$). The influence between genes can either

be activating ($w_{i,j} > 0$), inhibiting ($w_{i,j} < 0$) or absent ($w_{i,j} = 0$). The matrix $w = (w_{i,j})$ is the adjacency matrix of the regulatory pathway.

The equilibrium regulatory influence on each gene is given by $S_\infty$, which evolves from a starting state $S(0)$. This equilibrium state is viable if $S(t)$ is either convergent or cyclic within $k^2$ timesteps, at which point the equilibrium concentrations are given by $f(S(k^2))$ if $S(t)$ converges, or $f(avg(S(p : k^2)))$, where $p$ is the period of the cycle and $f$ maps regulatory influence to gene-product concentration. The simplest mapping corresponds to $f(S) = 1 + S$ so that down-regulation corresponds to a concentration of $0$, no regulation corresponds to basal transcription of $1$, and up-regulation corresponds to $2$. The scalar quantities for each case can be parameterized within the $f$ mapping.

To determine the *discrete* phenotype given an allele $M$, first the adjacency matrix $w$ using Equation 1 is computed. Then, $S_i$ is computed for $k^2$ time steps from the start state (e.g. $S(0) = [1, -1, -1, ..., -1]$), depicted in Figure 8. If $S$ converges or is cyclic within $k^2$ steps, then $S_\infty$ is the equilibrium regulatory state.

### 2.2.2 Viability

Lynch employed a simple viability constraint in his evolutionary analysis of pathways that required all genes to be regulated [3, ]. Intermediate transcription factors could be initiator signals for other pathways downstream, and so the loss of regulation of an intermediate gene which encodes a transcription factor would lead to the loss of the downstream pathway as well. Such a scenario would be considered fatal to the cell.

This viability criterion is imposed on the aforementioned *discrete* phenotype function. The calculation of viability is simple: the pathway is nonviable if any row in the pathway genotype is all zeros (no binding sites exist for the gene), and viable otherwise. If a pathway is not viable, then it is considered invalid.

## 2.3 Fitness

In this work, fitness is a function of the pathway equilibrium concentrations, given by the *discrete* equilibrium method, or a topological property of the pathway. Since the equilib-

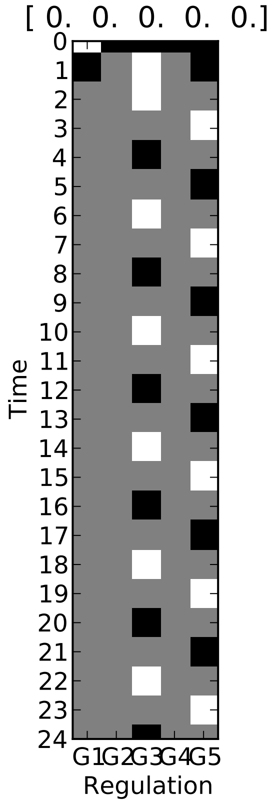


**Figure 8.** The time evolution of the example pathway using the *discrete* method. White signifies up-regulation, black down-regulation, and gray no regulation. The equilibrium concentrations for the gene-products can be found by averaging the cyclic behavior, which yields no regulation (the zero vector on top of the figure).

rium is given by a $k$-length binary array, we can classify genotypes by their associated $k$-length array into 'phenotype classes.' Genotypes in the same phenotype class are neutral with respect to one another.

Fitness is modeled under three scenarios:

1. *viability* - requires that all genes in the pathway are regulated, given by `viability`$(M)$, 0 otherwise.

2. *abstract phenotype* - each phenotype class (out of $2^k$ possible classes) is assigned a random fitness value from a uniform distribution. The fitness of a genotype is assigned based on its phenotype class. Each simulation regenerates the phenotype to

fitness mapping.

3. *pathway properties* - for a given pathway genotype $M$,

$$fitness_{prop}(M) = \texttt{viability}(M)|1.0 - s(L_{prop} - prop(M))|,$$

where $0 \leq L_{prop}, prop(M), s \leq 1$. The variable $s$ is a scaling factor which represents the strength of selection contributed by deviation from the optimal property $L_{prop}$. While it is not biologically reasonable to assume selection operates on topological properties of pathways alone, many studies have posited that redundancy, degree, or topology are driven by adaptive forces or that they are the result of some gradient ascent in fitness space. If this is the case, then there must be some contributing factor that these properties play in the overall fitness of the individual. This contributing factor is modeled as $s$, which may be insignificant ($s = 10^{-6}$) to substantial ($s = 1$). Therefore, $fitness_{prop}(M) \in \{0\} \cup [s, 1]$, since $\texttt{viability}(M)$ may equal 0. In this study, we examine the effect of selection on the average degree (*Degree*) of a pathway and the average number of redundantly regulated genes in the pathway (*Redundancy*).

# 3 Evolving pathways

## 3.1 Mutation

Since our model deals only with the non-coding mutations that effect pathway structure, pertinent mutations occur in the regulatory regions of the gene. Base pair mutations within regulatory regions either have no effect, remove a binding site, or result in the gain of a binding site. Lynch proposed formulas for the loss and gain rates of binding sites ($\mu_l$ and $\mu_g$ respectively) [3]. The loss rate is the per base pair mutation rate $u$ times the length of a binding site $n$. The gain rate is given by $Lnu/4^n$, where $L$ is the length of DNA that may harbor binding sites. The ratio of these rates $\alpha = \mu_l/\mu_g$ scales with the size of regulatory regions. Lynch showed that $\alpha$ is a function of the length of a binding site $n$ and the length of regulatory substrate per gene $L$: $\alpha = 4^n/L$.

Lynch recently calculated estimates of the per base pair mutation rate for organisms across all major phlya [6]. Most mutation rates range between $10^{-8}$ and $10^{-10}$, although viruses have extremely accelerated rates around $10^{-4}$. Therefore, if the binding site length is 10 bp, the loss rate of binding sites ranges from $10^{-7}$ to $10^{-9}$. $\alpha$ ranges from $10^{-3}$ in mammals to $10^{4}$ in bacteria, and so the gain rate has a wide spread of $10^{-4}$ to $10^{-13}$.

Modeling a mutation is straightforward. For a pathway allele $M$, the number of potential gain sites, $gain(M)$, is the count of zero values in the matrix $M$ and the number of loss sites, $loss(M)$, is the count of the number of non-zero entries. The total number of random loss mutations for the allele can be calculated using the binomial distribution, where $freq(M)$ is the frequency of allele $M$ in the population, and $u_\ell$ and $u_g$ are the loss and gain rates of binding sites:

$$\#_{losses} = binomial(Nfreq(M), loss(M)u_\ell)$$

And similarly the number of gain mutations:

$$\#_{gains} = binomial(Nfreq(M), gain(M)u_g)$$

Once the number of losses has been determined for a given network allele, $\#_{losses}$ mutant variants of $M$ are constructed by randomly (uniformly) selecting a binding site and removing it (setting $b_{i,j}$ to 0). This process may introduce new alleles into the population; however, not all mutant variants may be unique.

Similarly, once the number of gains has been determined, $\#_{gains}$ mutant variants of $M$ are constructed by randomly selecting an empty binding site and setting it equal to a random gene index in the pathway. The determination of an inhibition versus activation is parameterized by $\rho$, the probability of creating an activation regulatory effect. In this work, we use $\rho = 1/2$.

To update the affinity of a gain mutation at binding site $b_{i,j}$:

$$b_{i,j} = s_\rho(uniform(0,1)) \times uniform_{int}(1,k)$$

$$s_\rho(x) = \begin{cases} 1 & : x < \rho \\ -1 & : x \geq \rho \end{cases}$$

## 3.2 Recombination

To perform a crossover on the pathway genotype, let $M_1$ and $M_2$ be parent alleles with a crossover event occurring in the $b_{i,j}$ region. Let $m$ be the $k \times g$ pathway matrix unraveled into a $kg$ length vector such that $M_{i,j} = m_{ig+j}$. A recombinant is given as $m = m_1[1 : ig + j - 1] + m_2[ig + j : kg]$, with $m[ig + j]$ sampled from the relevant distributions in Figure 6. Disregarding redundancy in the discretized region does not preclude redundantly regulated recombinants to form, it only precludes redundancy within a position on the allele matrix. To avoid this issue altogether, it is possible to minimize $L/g$ by more finely discretizing the regulatory region (increasing $g$).

To perform a crossover between gene $i$ and gene $i + 1$, steps are taken as before to construct the parental $kg$ length vectors. Then, a recombinant is given by $m = m_1[1 : ig + g - 1] + m_2[(i + 1)g : kg]$.

## 3.3 Population simulations

Population genetic simulations were used to understand the combined effect of mutation, population size, and fitness on pathway evolution. In these experiments, an initially monomorphic population of pathways evolves, where we assume the population to be haploid, panmictic, and constant in size.

Given a population size $N$, generation time $G$, and mutation ratio $\alpha$, the population simulations performed in this study used the following method. First, a seed pathway genotype is randomly generated in addition to a $k$-length binary input signal, where $k$ is the number of genes in the pathway. The $k$-length binary input signal can be interpreted as the 'environmental input' to the regulatory pathway. The random pathway generated as the genotype is guaranteed to have $cM$ binding sites occupied, where $M$ is the maximum number of binding sites and $c$ is a proportion ($c = 0.5$ in all experiments). The input signal is used only for the simulations with fitness. A monomorphic (seed genotype) population of size $N$ is then evolved for $G$ generations. Results for complexity properties from a simulation are weighted averages of allele properties based on their frequency. Alleles are sampled after a burn in period of $G/2$, which allows for the population to reach steady

state. We developed custom code written in Python/C to handle population simulations.

# 4 Data provenance

We curated binding site data on three organisms, collecting for each known binding site the affiliated TF, affected TU, and distance from TSS. Using these properties of each binding site, we calculated the regulatory complexity properties as averages over reporting TFs (TFs present in the data):

- Avg TFBS = # TFBS per TU
- Avg Degree = # unique TFs per TU
- Avg Multiplicity = (average #TFBS per TF) per TU
- Avg Redundancy = Proportion of TUs with degree greater than 1

## 4.1 *E. coli*

We downloaded the *E. coli* TFBS data on 7/15/2011 from RegulonDB [7]. The TFBS flat file provides information on each known BS, its affiliated TU, and the distance from transcription start site.

## 4.2 *C. elegans* and *D. melanogaster*

We downloaded the BS information for *C. elegans* and *D. melanogaster* from modENCODE on 7/4/2011 using the provided web services for both genes and TFBS [8, 9]. Genome wide ChIP-chip experiments have matched several TFs to their binding sites throughout the *C. elegans* and *D. melangoster* genomes. However, the effect of each of these binding sites are not well known on downstream targets. To build a conservative estimate, we associated binding sites identified using ChIP-chip experiments with the closest downstream TU target and disregarded binding sites in introns, only using binding sites in intergenic regions between gene clusters, where overlapping genes were considered a single TU.

## 4.3 *S. cerevisiae*

We downloaded the TFBS on *S. cerevisiae*from SwissRegulonDB [10]. The flat file download provides a GFF file which we parsed using custom Python code.

## 4.4 *A. thaliana*

We downloaded the TFBS for *A. thaliana*from agris-AtcisDB (http://arabidopsis.med.ohio-state.edu/AtcisDB/) [11]. Non-coding regions which may harbor more binding sites were trucated at 3,000 bp.

## 4.5 On the quantity of data

Despite the lower number of TFs sampled in *C. elegans* and *D. melanogaster* in comparison to the other organisms, the distribution of TFBS count per TU matches that of *S. cerevisiae* and *E. coli K12*, following a scale-free pattern (see Fig 1A in the main text). The frequency of intergenic regions harboring a given amount of binding sites log linearly decreases as the number of binding sites increase. We observe this pattern regardless of the number of transcription factors, the number of intergenic regions, or the length of the intergenic regions. The data repository for binding sites in *A. thaliana* constrained upstream intergenic regions to be at most 3,000 bp, although larger upstream regions exist in the genome. We suspect that this truncation of intergenic regions in the *A. thaliana* data repository might skew the distribution of TFBS count/TU from that of the other organisms. Notwithstanding this truncation, the correlation of substrate length versus number of binding sites in the *A. thaliana* genome shows a very strong correlation ($r = 0.89$).

# References


1. Erb I, van Nimwegen E: **Statistical features of yeast's transcriptional regulatory code**. *IEEE Proc. ICCSB* 2006, **1**:111–118.

2. Bryne JC, Valen E, Tang MHE, Marstrand T, Winther O, da Piedade I, Krogh A, Lenhard B, Sandelin A: **JASPAR, the open access database of transcription factor-binding profiles: new content and tools in the 2008 update**. *Nucleic Acids Research* 2008, **36**(Database issue):D102–6, [[http://nar.oxfordjournals.org/content/36/suppl$_1/D102.long$]].

3. Lynch M: **The evolution of genetic networks by non-adaptive processes**. *Nature Reviews Genetics* 2007, **8**(10):803–13.

4. Ciliberti S, Martin OC, Wagner A: **Innovation and robustness in complex regulatory gene networks**. *Proc Natl Acad Sci USA* 2007, **104**(34):13591–6.

5. Martin OC, Wagner A: **Effects of recombination on complex regulatory circuits**. *Genetics* 2009, **183**(2):673–84.

6. Lynch M: **Evolution of the mutation rate**. *Trends Genet* 2010, **26**(8):345–52.

7. Gama-Castro S, Salgado H, Peralta-Gil M, Santos-Zavaleta A, Muñiz-Rascado L, Solano-Lira H, Jimenez-Jacinto V, Weiss V, García-Sotelo JS, López-Fuentes A, Porrón-Sotelo L, Alquicira-Hernández S, Medina-Rivera A, Martínez-Flores I, Alquicira-Hernández K, Martínez-Adame R, Bonavides-Martínez C, Miranda-Ríos J, Huerta AM, Mendoza-Vargas A, Collado-Torres L, Taboada B, Vega-Alvarado L, Olvera M, Olvera L, Grande R, Morett E, Collado-Vides J: **RegulonDB version 7.0: transcriptional regulation of Escherichia coli K-12 integrated within genetic sensory response units (Gensor Units)**. *Nucleic Acids Research* 2011, **39**(Database issue):D98–105, [[http://nar.oxfordjournals.org/content/39/suppl$_1/D98.long$]].

8. Gerstein MB, Lu ZJ, Nostrand ELV, Cheng C, Arshinoff BI, Liu T, Yip KY, Robilotto R, Rechtsteiner A, Ikegami K, Alves P, Chateigner A, Perry M, Morris M, Auerbach RK, Feng

X, Leng J, Vielle A, Niu W, Rhrissorrakrai K, Agarwal A, Alexander RP, Barber G, Brdlik CM, Brennan J, Brouillet JJ, Carr A, Cheung MS, Clawson H, Contrino S, Dannenberg LO, Dernburg AF, Desai A, Dick L, Dose AC, Du J, Egelhofer T, Ercan S, Euskirchen G, Ewing B, Feingold EA, Gassmann R, Good PJ, Green P, Gullier F, Gutwein M, Guyer MS, Habegger L, Han T, Henikoff JG, Henz SR, Hinrichs A, Holster H, Hyman T, Iniguez AL, Janette J, Jensen M, Kato M, Kent WJ, Kephart E, Khivansara V, Khurana E, Kim JK, Kolasinska-Zwierz P, Lai EC, Latorre I, Leahey A, Lewis S, Lloyd P, Lochovsky L, Lowdon RF, Lubling Y, Lyne R, Maccoss M, Mackowiak SD, Mangone M, Mckay S, Mecenas D, Merrihew G, Miller DM, Muroyama A, Murray JI, Ooi SL, Pham H, Phippen T, Preston EA, Rajewsky N, Ratsch G, Rosenbaum H, Rozowsky J, Rutherford K, Ruzanov P, Sarov M, Sasidharan R, Sboner A, Scheid P, Segal E, Shin H, Shou C, Slack FJ, Slightam C, Smith R, Spencer WC, Stinson EO, Taing S, Takasaki T, Vafeados D, Voronina K, Wang G, Washington NL, Whittle CM, Wu B, Yan KK, Zeller G, Zha Z, Zhong M, Zhou X, Ahringer J, Strome S, Gunsalus KC, Micklem G, Liu XS, Reinke V, Kim SK, Hillier LW, Henikoff S, Piano F, Snyder M, Stein L, Lieb JD, Waterston RH: **Integrative Analysis of the Caenorhabditis elegans Genome by the modENCODE Project**. *Science* 2010, **330**(6012):1775–1787.

9. Roy S, Ernst J, Kharchenko PV, Kheradpour P, Negre N, Eaton ML, Landolin JM, Bristow CA, Ma L, Lin MF, Washietl S, Arshinoff BI, Ay F, Meyer PE, Robine N, Washington NL, Stefano LD, Berezikov E, Brown CD, Candeias R, Carlson JW, Carr A, Jungreis I, Marbach D, Sealfon R, Tolstorukov MY, Will S, Alekseyenko AA, Artieri C, Booth BW, Brooks AN, Dai Q, Davis CA, Duff MO, Feng X, Gorchakov AA, Gu T, Henikoff JG, Kapranov P, Li R, Macalpine HK, Malone J, Minoda A, Nordman J, Okamura K, Perry M, Powell SK, Riddle NC, Sakai A, Samsonova A, Sandler JE, Schwartz YB, Sher N, Spokony R, Sturgill D, Baren MV, Wan KH, Yang L, Yu C, Feingold E, Good P, Guyer M, Lowdon R, Ahmad K, Andrews J, Berger B, Brenner SE, Brent MR, Cherbas L, Elgin SCR, Gingeras TR, Grossman R, Hoskins RA, Kaufman TC, Kent W, Kuroda MI, Orr-Weaver T, Perrimon N, Pirrotta V, Posakony JW, Ren B, Russell S, Cherbas P, Graveley BR, Lewis S, Micklem G, Oliver B, Park PJ, Celniker SE, Henikoff S, Karpen GH, Lai EC, Macalpine

DM, Stein LD, White KP, Kellis M, Acevedo D, Auburn R, Barber G, Bellen HJ, Bishop EP, Bryson TD, Chateigner A, Chen J, Clawson H, Comstock CLG, Contrino S, Denapoli LC, Ding Q, Dobin A, Domanus MH, Drenkow J, Dudoit S, Dumais J, Eng T, Fagegaltier D, Gadel SE, Ghosh S, Guillier F, Hanley D, Hannon GJ, Hansen KD, Heinz E, Hinrichs AS, Hirst M, Jha S, Jiang L, Jung YL, Kashevsky H, Kennedy CD, Kephart ET, Langton L, Lee OK, Li S, Li Z, Lin W, Linder-Basso D, Lloyd P, Lyne R, Marchetti SE, Marra M, Mattiuzzo NR, Mckay S, Meyer F, Miller D, Miller SW, Moore RA, Morrison CA, Prinz JA, Rooks M, Moore R, Rutherford KM, Ruzanov P, Scheftner DA, Senderowicz L, Shah PK, Shanower G, Smith R, Stinson EO, Suchy S, Tenney AE, Tian F, Venken KJT, Wang H, White R, Wilkening J, Willingham AT, Zaleski C, Zha Z, Zhang D, Zhao Y, Zieba J: **Identification of Functional Elements and Regulatory Circuits by Drosophila modENCODE**. *Science* 2010, **330**(6012):1787–1797.

10. Pachkov M, Erb I, Molina N, van Nimwegen E: **SwissRegulon: a database of genome-wide annotations of regulatory sites**. *Nucleic Acids Research* 2007, **35**(Database):D127–D131.

11. Yilmaz A, Mejia-Guerra MK, Kurz K, Liang X, Welch L, Grotewold E: **AGRIS: the Arabidopsis Gene Regulatory Information Server, an update**. *Nucleic Acids Research* 2011, **39**(Database):D1118–D1122.
